# Genotype determines *Arbutus unedo* L. physiological and metabolomic responses to drought and recovery

**DOI:** 10.3389/fpls.2022.1011542

**Published:** 2022-11-22

**Authors:** João Martins, Pierre Pétriacq, Amélie Flandin, Aurelio Gómez-Cadenas, Pedro Monteiro, Glória Pinto, Jorge Canhoto

**Affiliations:** ^1^ Centre for Functional Ecology, Department of Life Sciences, University of Coimbra, Calçada Martim de Freitas, Coimbra, Portugal; ^2^ UMR BFP, University Bordeaux, INRAE, Villenave d’Ornon, France; ^3^ Bordeaux Metabolome, MetaboHUB, PHENOME-EMPHASIS, Villenave d’Ornon, France; ^4^ Departamento de Ciencias Agrarias y del Medio Natural, Universitat Jaume I, Castelló, Spain; ^5^ Centre for Environmental and Marine Studies, Department of Biology, University of Aveiro, Aveiro, Portugal

**Keywords:** drought, phenolic compounds, micropropagation, plant hormones, strawberry tree

## Abstract

Strawberry tree (*Arbutus unedo*) is a small resilient species with a circum-Mediterranean distribution, high ecological relevance in southern European forests and with several economical applications. As most orchards are usually installed on marginal lands where plants usually face severe drought, selecting plants that can better cope with water restriction is critical, and a better understanding of the tolerance mechanisms is required. Strawberry tree plants under drought follow a typical isohydric strategy, by limiting transpiration through stomata closure. However, the contribution of genotype and its bio-geographic origin on plant performance needs clarification, as well as the involvement of a specific metabolic reactions associated with the mechanical response. To test this hypothesis, several eco-physiological and biochemical parameters were assessed on different genotypes, and the metabolic profiles studied, including important stress-related phytohormones, on plants under different water regimes (plants watered to 70% and 18% field capacity) and a recovery assay. A contrasting drought tolerance was found in plants from different genotypes, associated with physiological and metabolic responses. Metabolomics revealed more than 500 metabolic features were differentially accumulated, including abscisic and salicylic acids, for the genotype with better performance under drought (A4). This genotype also recovered faster when the imposed stress was interrupted, thus indicating the relevance of metabolic adaptation under water deficit conditions. By correlating carbon assimilation with identified metabolites, some proved to be satisfactory predictors of plant performance under drought and might be used for marker assisted breeding. Therefore, our study proves the importance of genotype as a major selection criterion of resistant plants to drought and provides empirical knowledge of the metabolic response involved. We also hypothesized the involvement of phenolics on response mechanisms under drought, which is worth to be explored to shed light on the metabolic pathways involved in plant response to water stress.

## 1 Introduction

Strawberry tree (*Arbutus unedo* L., Ericaceae) is an evergreen species quite tolerant to a wide range of temperatures, that grows on rocky and dry soils around the Mediterranean basin and Atlantic coast of Portugal, Spain, France, and Ireland (Torres et al., 2002). Due to its ability to colonise marginal lands and resprout after forest fires, it is a key species in the Mediterranean biome and the forest ecosystems of South Europe by avoiding the spread of invasive species and desertification (Martins et al., 2021c). The bioactive compounds (e.g., arbutin and hydroquinone), along with their round edible berries, are the primary income for farmers. Due to its great economic potential and increasing demand, the production area has been constantly growing in recent years, especially on marginal lands with poor soils and scarce water where most species hardly thrive, being particularly exposed to drought conditions, which is one of the most severe environmental stresses affecting plant growth and development (Seleiman et al., 2021), broadly impacting physiological and biochemical processes. Mediterranean species such as *A. unedo* are drought-tolerant (Castell and Terradas, 1994; Munné-Bosch and Peñuelas, 2004; Martins et al., 2021b), but in the context of global climate change, which predicts difficult conditions for southern Europe and the Mediterranean region, even the most water-stress tolerant species may exceed their capacity for acclimatation and drought resistance (Nardini et al., 2014), especially sclerophyllous Mediterranean species (Bussotti et al., 2014).

Plants have developed a plethora of response mechanisms to drought, that vary significantly depending on the species, but also at provenance, genotype, and organ levels, highlighting the complexity of drought response among trees (de Simón et al., 2017). The relevance of genotype in response to drought conditions has been found for several species, as altered metabolic levels also link to different genotypes of *Cicer arietinum* (Khan et al., 2019) and *Sesamum indicum* (You et al., 2019). In species such as *Arabidopsis thaliana*, *Oryza sativa*, *Solanum lycopersicum* and *Salix* sp., drought stress triggers downstream pathways that involve plant hormones like abscisic (ABA), jasmonic (JA) and salicylic (SA) acids (Khan et al., 2015; Vishwakarma et al., 2017; Yang et al., 2019), and initiates the biosynthesis of different types of protective secondary metabolites like phenolic acids, flavonoids, and proline (Hayat et al., 2012; Sarker and Oba, 2018). These responses minimise the adverse effects of drought by reducing water loss and oxidative stress (Fàbregas and Fernie, 2019; Yadav et al., 2021). ABA plays a key role in the response mechanism by regulating stomatal opening and gene expression (Takahashi et al., 2020). Flavonoids have also been linked to plant protection mechanisms against abiotic stress due to their broad spectrum of biological activities that includes signaling, auxin transport, pigmentation, and modulation of reactive oxygen species (ROS), either by ROS scavenge and inhibition or through the activation of antioxidant enzymes (Laoué et al., 2022). In fact, the involvement of flavonoids in drought response, such as quercetin, myricetin and kaempferol was reported in several species: *Fraxinus ornus* (Fini et al., 2012), *Populus* spp. (Popović et al., 2016), *Quercus ilex* (Rivas-Ubach et al., 2014), and *Vitis vinifera* (Griesser et al., 2015). Thus, the comprehension of metabolic pathways leading to the synthesis of water stress-related compounds is crucial for a better grasp of drought resistance physiological mechanisms.

Research conducted to date have highlighted the conservative water use strategy of strawberry tree, an isohydric resprouter with tight stomatal control able to maintain a low gas exchange rate, which proves useful during long drought stress periods, and with high phenotypic plasticity that is in part under the control of genetic traits (Martins et al., 2019; Martins et al., 2021b). However, the factors governing the resistance mechanism are not yet fully understood, and a more detailed analysis is necessary. The role of the genotype and plant provenience needs clarification as previously published studies are not consistent. Data obtained by Vasques et al. (2013) suggested a correlation between the bio-geographic origin of the plants with their performance under drought, but a different study (Martins et al., 2021b) found no correlation between these two factors. Furthermore, there remains a paucity of evidence on the involvement of a metabolic response associated with drought in strawberry tree. Metabolomics data can also serve as a phenotype predictor of plant performance under drought stress, considerably reducing the time required in breeding programmes (Sprenger et al., 2018; Dussarrat et al., 2021). As pant selection usually greatly relies on genetic markers which presupposes in-depth knowledge of population genetics, the identification of metabolomic markers is essential on a species such as strawberry tree, that lacks genetic studies.

Therefore, the present study investigated the metabolic responses to drought in strawberry tree genotypes with different geographic origins. This work provides the first metabolic profiling under drought in this species, that along with physiological data will be an important empirical basis for plant selection and breeding. We also shed light on acclimation by studying the drought and recovery and prove the metabolomic response is genotype dependent. Finally, we hypothesized the involvement of phenolics on protection mechanisms under drought, and by correlating carbon assimilation with identified metabolites, we provide a basis for marker assisted breeding towards drought resistance.

## 2 Methods

### 2.1 Micropropagation

Four genotypes from different provenances were selected: A1 and A3 from the centre region of Portugal, from an area with a high (>1000 mm) and a medium (500-1000 mm) average rainfall, respectively, and A2 and A4 from the south region, both from an area with a low (<500 mm) average rainfall (**Figure S1A**). Genotype A1 was established *in vitro* from an adult tree, whereas genotypes A2, A3 and A4 from seedlings, as described by (Martins et al., 2019). For axillary shoot proliferation shoots were inoculated in Anderson Rhododendron medium (Anderson, 1980) with 6-benzylaminopurine (2 mg L^-1^, Sigma-Aldrich, St. Louis, MO, USA), sucrose (3%, w/v, Duchefa) and agar (0.6%, w/v, Duchefa). The pH was adjusted to 5.7 using KOH or HCl diluted solutions (0.01 M – 1 M), and the culture medium was autoclaved at 121 °C for 20 min (800–1100 g cm^-2^ gel strength after autoclaving). The culture was done on Microbox plastic containers (O118/80+OD118 with white filter, SacO_2_, Deinze, Belgium) with 100 mL of media, on a growth chamber at a 16-h photoperiod, an irradiance of 15 - 20 µmol m^-2^ s^-1^ (cool-white fluorescent lamps), and a temperature of 25 °C, with culture intervals of 8 weeks.

For rooting, 3 cm long shoots were dipped on indole-3-butyric acid (IBA; 1 gr L^-1^, Sigma-Aldrich) for 30 seconds and placed on covered containers with perlite (Siro, Mira, Portugal), on a walk-in growth chamber (FitoClima 10000 HP, Aralab, Rio de Mouro, Portugal) under 16-h photoperiod at 25 °C, 70% humidity and 250 µmol m^-2^ s^-1^ irradiance). The cover was gradually removed, and after a month, plants were transferred to individual containers (1700 cm^3^) with a substrate composed of peat (30-0; Siro) and perlite (3:1; v/v). Plants were kept under these conditions for two years, watered to 70-80% field capacity. A fertiliser (NPK, 12-12-17; Siro) was applied after one year.

### 2.2 Water stress

#### 2.2.1 Drought stress assay and experimental design

Two-year-old plants kept on the conditions described before were placed under three water regimes using the gravimetric method: WW – well-watered (watered to 70% field capacity), WS – water stress (watered to 18% field capacity) and RC – recover (watered to 18% and then to 70% field capacity) (**Figure S1B**). Three sampling points were as followed: t1 (10 days) and t2 (20 days) for WS groups and t3 (20 days with 18% field capacity plus 5 days with 70%) for RC groups. A WW group (control) was also sampled at each time point. Plant performance was evaluated on the four genotypes based on physiological (gas exchange and water status), biochemical (phenols, ortho-phenols, flavonoids, proline, chlorophyll a and b, carotenoids and anthocyanins) and metabolomic parameters (untargeted and targeted). 5-7 plants from each of the four genotypes were sampled for WW and RC groups and 6-7 plants for WS group at each sampling point (t1, t2 and t3). For biochemical and metabolomic analysis, apical leaves were collected and flash-frozen in liquid nitrogen to rapidly arrest metabolic activity, ground in liquid nitrogen with a mortar and pestle and transferred to 2 mL microtubes. Samples for metabolomic analysis were freeze-dried for 48 h (Coolsafe, Labogene, Lynge, Denmark). Biochemical analysis was carried out at t2 (n=6-7) and t3 (n=5-7). Untargeted metabolomic analysis was carried out at t2 (n=4) and t3 (n=5). Targeted metabolomic analysis (abscisic, jasmonic and salicylic acids) was also carried out at t2 (n=3) and t3 (n=3), both with genotypes A1 and A4 only, that were selected based on the physiological and metabolic data.

#### 2.2.2 Gas exchange and plant water status

*In situ* leaf gas exchange measurements (net CO_2_ assimilation rate: A, transpiration rate: E, stomatal conductance: gs, and the intercellular CO_2_ concentration: ci) were performed using a portable infrared gas analyser (LCpro*+*, ADC, Hoddesdon, UK), operating in open mode and under the following conditions: photosynthetic photon flux density: 350 µmol m^-2^ s^-1^; air flux: 200 mol s^-1^; block temperature: 25°C; and atmospheric CO_2_ and H_2_O concentration. Data were recorded when the measured parameters were stable (2–6 min). Water potential (Ψ) was measured on the main stem of plants (cut 10 cm from the apical meristem) with a Scholander-type pressure chamber (PMS Instrument Co., Albany, OR, USA).

### 2.3 Biochemical analysis

#### 2.3.1 Phenols

Total phenols in the extracts were estimated according to (Attard, 2013). Briefly, 1.5 mL methanol (70%, v/v) was added to 40 mg of frozen plant material. Samples were kept on an orbital shaker at 700 rpm, 25°C for 1 hour and centrifuged for 15 min, 10000 g, 4°C. The supernatant was collected, and the extraction was repeated thrice. The final volume was adjusted to 10 mL with methanol (70%, v/v). For quantification, 20 μL of the sample was mixed with 90 μL distilled water, 10 μL Folin-Ciocalteau reagent solution and 80 μL sodium carbonate (7%, w/v). After 2-hour incubation in the dark, the absorbance was read at 520 nm in a microplate reader. The concentration of phenols was determined as gallic acid equivalents from a standard curve (0-250 μg, y = 5.2429x + 0.0541, R² = 0.9998).

#### 2.3.2 Ortho-phenols

Ortho-phenols were quantified according to (Maestro Durán et al., 1991). Methanolic extracts were prepared as described before. Quantification was done by mixing 160 μL of sample with 40 μL sodium molybdate (5%, w/v). After 15 min incubating in the dark the absorbance was read at 370 nm in a microplate reader. Ortho-phenols concentration was determined as equivalents of gallic acid from a standard curve (0-250 μg, y = 5.6032x + 0.0503, R² = 0.9999).

#### 2.3.3 Flavonoids

Total flavonoids were estimated according to (Zhishen et al., 1999). Methanolic extracts were prepared as described before, and quantification was done by mixing 60 μL of the sample with 28 μL sodium nitrite (5%, w/v). After 6 min incubating in the dark, 28 μL aluminium chloride (10%, w/v) was added and samples were incubated in the dark for 6 min. Finally, 120 μL sodium hydroxide (4%, w/v) were added to the mixture and absorbance was read at 510 nm in a microplate reader. The concentration of flavonoids was determined as equivalents of catechin from a standard curve (0-125 μg, y = 8.8685x + 0.0545, R² = 0.9997).

#### 2.3.4 Proline

Total proline in the extracts was estimated according to (Bates et al., 1973). Briefly, 750 µL of sulfosalicylic acid (3%, v/v) was added to 50 mg of frozen plant material and centrifuged for 10 min, 10000 g, 4°C. 500 µL of supernatant was collected, and 500 µL of ninhydrin and glacial acetic acid were added. Samples were then incubated at 100°C for 1 hour and cooled on ice. Finally, the reaction mixture was extracted with toluene, and the absorbance of the chromophore-containing toluene was read at 520 nm in a microplate reader. Proline concentration was determined from a L-Proline standard curve (0-15 μg, y = 0.0799x + 0.0056, R² = 0.9998).

#### 2.3.5 Chlorophyll and carotenoids

Total chlorophyll and carotenoids in the extracts was estimated according to (Sims and Gamon, 2002). 50 mg of frozen plant material was ground in 2 mL of acetone:Tris buffer, 50mM, pH 7.8 (80:20) and centrifuged for 5 min (10000 g, 4°C). Supernatant was collected and extraction was repeated with 3 mL acetone:Tris. Finally, acetone:Tris was added to the supernatants to obtain a final volume of 6 mL. Samples were kept on ice and protected from light during the entire process. The absorbance of supernatants was read at 470nm, 537nm, 647nm and 663nm on a UV-Vis spectrophotometer. acetone:Tris buffer, 50mM, pH 7.8 (80:20) was used as blank. Chlorophyll *a* (Chla), Chlorophyll *b* (Chlb) and Carotenoids (Car) contents were calculated according to the following equations: Chla = 0,01373 * A663 – 0,000897 * A537 – 0,003046 * A647; Chlb = 0,02405 * A647 – 0,004305 * A537 –0,005507 * A663; Car = ((A470 – (17,1 * (Chla + Chlb) – 9,479 * Anthocyanins))/119,26 and Anthocyanins = 0,08173 * A537 – 0,00697 * A647 – 0,002228 * A663.

#### 2.3.5 Anthocyanins

*In situ* total anthocyanins were estimated following the protocol of (Close et al., 2004), with minor adaptations. 50 mg of frozen plant material was ground in 2 mL of acidified ethanol (ethanol:HCl, 99:1, v/v). The homogenate was immersed in boiling water for 90 seconds, and kept in the dark for 24 hours at 4°C. After centrifugation (10000 g, 10 min, at 4°C), absorbance was measured using a UV-Vis spectrophotometer at 530 and 657 nm. The formula A530 – 0.25 * A657 was used to calculate anthocyanins content.

### 2.4 Metabolomic analysis

#### 2.4.1 Untargeted metabolic profiling

10 milligrams of each replicated lyophilised sample were weighed into 1.1 mL-micronic tubes (MP32033L, Micronic, Lelystad, Netherlands), randomised onto a 96-micronic rack (MPW51001BC6, Micronic), then capped using a robotised capper-decapper (Decapper 193000/00, Hamilton, Bienne, Switzerland). Each rack also contained an empty tube corresponding to the extraction blank. The resulting micronics were then stored at -80°C. Metabolites extraction was conducted on three or four biologically replicated leaf samples (n = 3-5) using a robotised extraction method developed at Bordeaux Metabolome Facility (https://metabolome.cgfb.u-bordeaux.fr/en, Villenave d’Ornon, France) as described by (Luna et al., 2020).

Untargeted metabolic profiling by UHPLC-LTQ-Orbitrap mass spectrometry (LCMS) was conducted using an Ultimate 3000 ultra-high-pressure liquid chromatography (UHPLC) system coupled to an LTQ-Orbitrap Elite mass spectrometer interfaced with an electrospray (ESI) ionisation source (ThermoScientific, Bremen, Germany) operating in negative ion mode as described previously (Luna et al., 2020). MS1 full scan detection of ions was performed by FTMS (50 - 1500 Da) at a resolution of 240k at 200 *m/z*. 10 QC samples and 5 blank extracts were injected to correct for mass spectrometer signal drift, and to filter out variables detected in blanks, respectively. MS2 Data Dependent Analysis (DDA) was also performed on QC sample to generate fragmentation information for further annotation with the following parameters: FTMS (50 - 1500 Da) at a resolution of 60k at 200 *m/z*; activation type, CID; isolation width, 1 Da; normalised collision energy, 35 eV; activation Q, 0.250; activation time, 10 ms). Phytohormone standards were also injected along with sample extracts for annotation purposes.

Raw LCMS data were processed using MS-DIAL v 4.60 (Tsugawa et al., 2015), yielding 12 282 RT-*m/z* features. MS-DIAL parameters were as follows: MS1, tolerance, 0.01 Da; MS2 tolerance, 0.025 Da; retention time begin, 0 min; retention time end, 18 min; minimum peak height, 10000; mass slice width, 0.1 Da; smoothing level, 3 scans; minimum peak width, 5 scans; sigma window value, 0.5. After data-cleaning (blank check, SN > 10, CV QC < 30%), 3953 variables were retained for further chemometrics. MS-DIAL annotation of metabolic features based on MS1 HRMS and MS2 fragmentation information was performed using the online library MSMS-Public-Neg-VS15.msp (36,848 records) with the following parameters: retention time tolerance, 100 min; accurate mass tolerance (MS1), 0.01 Da; accurate mass tolerance (MS2), 0.05 Da; identification score cut off, 80%. Putative annotation of differentially expressed metabolites resulted from MS-DIAL screening of the MS1 detected exact HR *m/z* and MS2 fragmentation patterns against multiple online databases (http://prime.psc.riken.jp/compms/msdial/main.html#MSP) (Tsugawa et al., 2015).

#### 2.4.2 Hormone quantification

Leaf content of abscisic acid (ABA), jasmonic acid (JA) and salicylic acid (SA) from A1 and A4 genotypes (WW, WS and RC) were analysed by UPLC MS/MS (ultra-performance liquid chromatography, tandem mass spectrometry) as described by (Durgbanshi et al., 2005) with slight modifications. For extraction, 2 mL of ultrapure water were added to 0.1 g of lyophilised leaf material and blended in a ball mill (MillMix20, Domel, Železniki, Slovenija). Water was spiked with 50 ng of [^2^H_6_]-ABA, [^13^C_6_]-SA and dihydrojasmonic acid. After centrifugation at 4000 g at 4°C for 10 min, supernatants were recovered, and pH adjusted to 3 with acetic acid (30%, v/v). All water extracts were partitioned twice against 2 mL of diethyl-ether and the organic layer was recovered and evaporated under vacuum in a centrifuge concentrator (Speed Vac, Jouan, Saint Herblain Cedex, France). The dried residue was resuspended in water: methanol (9:1, v/v) by gentle sonication. The resulting solution was filtered through a 0.22 μm polytetrafluoroethylene membrane syringe filter (Albet S.A., Barcelona, Spain) and injected into an ultra-performance liquid chromatography system (Acquity SDS, Waters Corp., Milford, MA, USA). Chromatographic separations were carried out on a reversed-phase C18 column (Luna, Omega, 50 × 2.1 mm, 1.6 μm particle size, Phenomenex, Madrid, Spain) using a methanol:water gradient [both supplemented with acetic acid (0.1%, v/v)] at a flow rate of 300 μL min^−1^. Hormones were quantified with a triple quadrupole mass spectrometer (Micromass, Manchester, UK) connected online to the output of the column though an orthogonal Z-spray electrospray ion source.

### 2.5 Statistical analysis

To compare genotypes under different water conditions, physiological and biochemical data were analysed by two-way ANOVA using GraphPad Prism (v. 8.4.3 for Windows, San Diego, CA, USA), followed by a Tukey´s multiple comparison test (P < 0.05). Values are given as means ± standard deviations. To evaluate the interaction and significance of the biochemical and physiological parameters on the analysed genotypes, Principal Component Analysis (PCA) and heatmaps were constructed using R software (version 4.0.3, R Foundation for Statistical Computing, Vienna, Austria (R Core Team, 2020). The PCA was performed using the prcomp function and the package ggbiplot (Vu, 2011). The heatmap was constructed with the Heatmap function and the package ComplexHeatmap (Gu et al., 2016). The dendrogram within the heatmap was calculated with Euclidean distance as similarity measure. Metabolomic data was normalised by weight (10 mg), then by median normalisation, cube-root transformation and Pareto scaling using MetaboAnalyst v. 5.0 (Pang et al., 2021), before statistical analyses. Heatmaps were constructed with the most significant different metabolites (one-way Anova, P < 0.01). Volcano plots were used to identify statistically significant variation on metabolites between control (WW) and treatment (WS and RC) groups (FC > 2, P < 0.05). To check for differently accumulated metabolites shared between genotypes, Venn diagrams were constructed on R software using the package VennDiagram (Chen, 2018). Finally, to putatively identify metabolomic markers, linear regression models were calculated using the lm function from R software, to predict net CO_2_ assimilation rates with metabolites relative concentration. Only identified metabolites with statistical differences between treatments were used for this analysis. Before the linear models were constructed, Cook’s distance was calculated to remove outliers, and Jarque-Bera and Durbin-Watson tests were performed to test the normality and independence of residuals, respectively.

## 3 Results

To study drought stress responses in strawberry tree at the physiological and biochemical levels, a time-course analysis was performed at three time points under different water regimes. Gas exchange parameters and carbon assimilation were measured along with plant water stratus. Several biochemical parameters were quantified and a metabolomic profile was obtained, including the quantification of stress related hormones (ABA, JA and SA). A multivariate analysis was carried out to identify patterns in the metabolic response do drought and a linear regression model was implemented to identify metabolic markers.

### 3.1 Physiological and biochemical analysis

**Figure 1:**
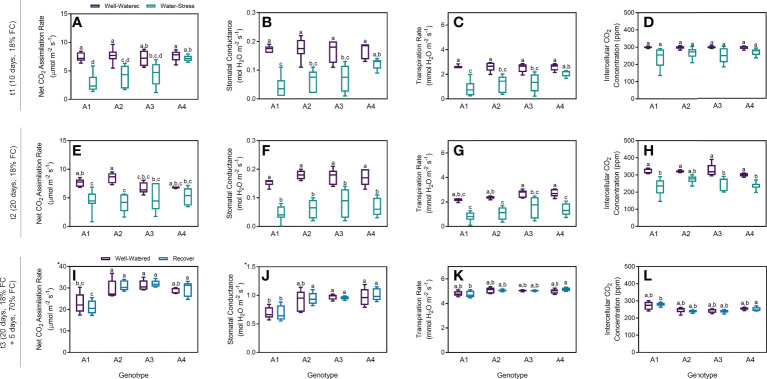
Physiological parameters measured on t1 (10 days), t2 (20 days) and t3 (20 + 5 days) on 4 genotypes (A1, A2, A3 and A4) and 3 water regimes (well-watered, water-stress and recover): net CO_2_ assimilation rate at t1 **(A)**, stomatal conductance at t1 **(B)**, transpiration rate at t1 **(C)**, intercellular CO_2_ concentration at t1 **(D)**, net CO_2_ assimilation rate at t2 **(E)**, stomatal conductance at t2 **(F)**, transpiration rate at t2 **(G)**, intercellular CO_2_ concentration at t2 **(H)**, net CO_2_ assimilation rate at t3 **(I)**, stomatal conductance at t3 **(J)**, transpiration rate at t3 **(K)**, and intercellular CO_2_ concentration at t3 **(L)**. Means ± SDs, n = 5-7, different letters indicate significant differences between treatments at *P ≤* 0.05 according to a Tukey’s multi comparison test.

Stomatal conductance was determined to estimate the movement of gases, and four parameters related to carbon dioxide uptake and water loss are presented to provide an overview of stomata aperture and carbon assimilation under different water regimes (**Figure 1**). Net CO_2_ assimilation rate decreased in plants under water stress after 10 days (t1), in genotypes A1 and A2 (**Figure 1A**). Although this tendence was observed on genotype A3, no statistical differences between the control and stress groups were found. Finally, no difference was observed for genotype A4. Moreover, plants under stress from genotype A4 had higher rates of net CO_2_ assimilation than those from genotypes A1 and A2 under stress (**Figure 1A**). Lower stomatal conductance and transpiration rate were also observed with statistical differences between genotypes A1, A2 and A3 (**Figures 1B, C**). Nevertheless, no statistical differences were found for intercellular CO_2_ concentration between well-watered (WW) and water stress (WS) groups, for the four genotypes tested (**Figure 1D**). As observed after t1, net CO_2_ assimilation rate was lower in plants under stress after 20 days (t2). Similarly, no statistical differences were observed on genotypes A3 and A4 between control and stress groups (**Figure 1E**). Moreover, stomatal conductance considerably decreased in plants under water restrictions, with statistical difference between WW and WS groups, in the four genotypes (**Figure 1F**), as well as transpiration rates (**Figure 1G**). The only exception was intercellular CO_2_ concentration on genotype A2 (**Figure 1H**). For the recovered plants, all the physiological parameters were very similar when compared to the control groups on the four genotypes tested (**Figures 1I–L**).

**Figure 2:**
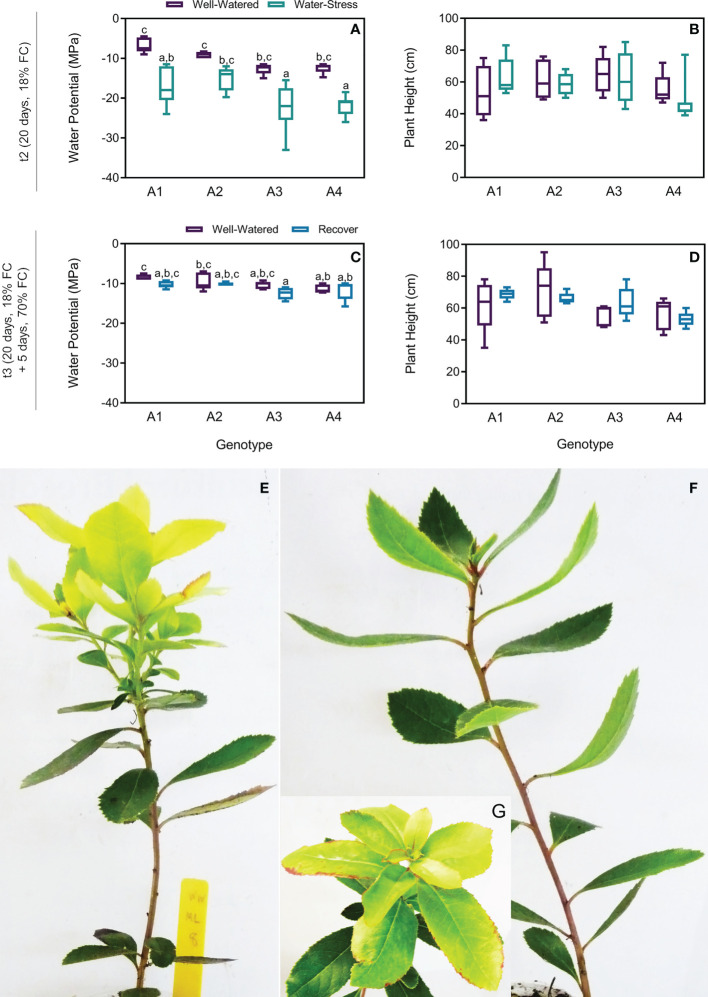
Water potential and plant height on t2 (20 days) and t3 (20 + 5 days) on 4 genotypes (A1, A2, A3 and A4) and 3 water regimes (well-watered, water-stress and recover) and morphological changes under drought: water potential on t2 **(A)**, plant height on t2 **(B)**, water potential on t3 **(C)**, plant height on t3 **(D)**, strawberry tree plant from genotype A1 under well-watered (WW) **(E)** and water-stress (WS) conditions **(F, G)**. Means ± SDs, n = 5-7, different letters indicate significant differences between treatments at *P ≤* 0.05 according to a Tukey’s multi comparison test.

To evaluate the effect of drought on plant water status and growth, stem water potential and plant height were measured. Although a tendency of decrease in the water potential was observed in plants under drought at t2, no statistical differences were found between treatments in the four genotypes (**Figure 2A**). Similarly, no differences were found on plant height (**Figure 2B**). In the recovered plants the water potential return to the basal level without differences between treatments (**Figure 2C**). Additionally, no differences were found in the recovered plants height (**Figure 2D**). In contrast with control plants (**Figure 2E**), plants under drought showed mild symptoms of drought, which included leaves curling (**Figure 2F**) and burning or scorching on edges of young leaves (**Figure 2G**). After 5 days (t3), recovered plants did not recover from these symptoms.

**Figure 3:**
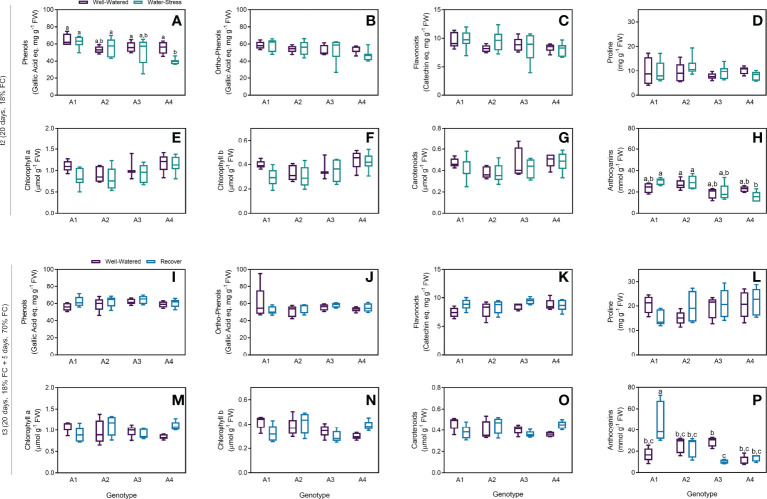
Biochemical parameters measured on t2 (20 days) and t3 (20 + 5 days) on 4 genotypes (A1, A2, A3 and A4) and 3 water regimes (well-watered, water-stress and recover): phenols on t2 **(A)**, ortho-phenols on t2 **(B)**, flavonoids on t2 **(C)**, proline on t2 **(D)**, chlorophyll a on t2 **(E)**, chlorophyll b on t2 **(F)**, carotenoids on t2 **(G)** and anthocyanins on t2 **(H)**, phenols on t3 **(I)**, ortho-phenols on t3 **(J)**, flavonoids on t3 **(K)**, proline on t3 **(L)**, chlorophyll a on t3 **(M)**, chlorophyll b on t3 **(N)**, carotenoids on t3 **(O)** and anthocyanins on t3 **(P)**. Means ± SDs, n = 5-7, different letters indicate significant differences between treatments at *P ≤* 0.05, according to a Tukey’s multi comparison test.

As an overview of the general biochemical response of strawberry tree plants under drought, several biochemical parameters usually involved in plant response to water deficit were quantified, including phenols and flavonoids equivalents, proline, chlorophylls, carotenoids and anthocyanins. In general, no differences were found for levels of phenols, ortho-phenols, flavonoids and proline between WW and WS groups (**Figures 3A–D**) after t2. A statistical difference was only found in genotype A4, with lower phenol concentrations for plants under stress (**Figure 3A**). Although the levels of chlorophyll a and b and carotenoids slightly decreased in plants under water stress, no statistical differences were found for all the samples (**Figures 3E–G**). On the other hand, the levels of anthocyanins increased in genotypes A1 and A2 under water stress, whereas a decrease was observed in A4 (**Figure 3H**). Nonetheless, no statistical differences were obtained between WW and WS groups regardless of the genotypes. For the recovered plants, all the parameters were very similar when compared to the control groups on the four genotypes tested (**Figures 3I–P**). The only exception was the higher amounts of anthocyanins in recovered plants from genotype A1 and lower in genotype A3, with statistical differences (**Figure 3P**). Although the levels of proline, chlorophyll a and b and carotenoids were lower in recovered plants from genotype A1, this tendency has no statistical significance (p-values = 0.33, 0.57, 0.78 and 0.76, respectively).

**Figure 4:**
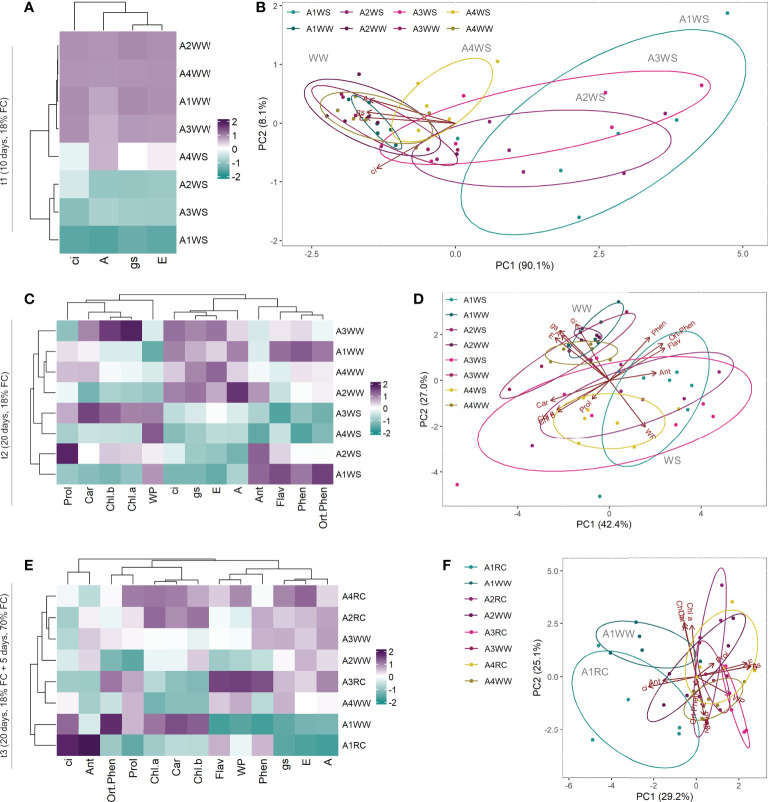
Heatmap and Principal Component Analysis with physiological and biochemical parameters measured on t1 (10 days), t2 (20 days) and t3 (20 + 5 days) on 4 genotypes (A1, A2, A3 and A4) and 3 water regimes (well-watered, water-stress and recover): heatmap on t1 **(A)**, PCA on t1 **(B)**, heatmap on t2 **(C)**, PCA on t2 **(D)**, heatmap on t3 **(E)** and PCA on t3 **(F)**. A, net CO_2_ assimilation rate; Ant, anthocyanins; Car, carotenoids; Chl.a, chlorophyll a; Chl.b, chlorophyll b; ci, intercellular CO_2_ concentration; E, transpiration rate; Flav, flavonoids; gs, stomatal conductance; Phen, phenols; Prol, proline; Ortho.Phen, ortho-phenols; WP, water potential.

To study the interaction and correlation between the measured variables and genotypes, a clustering heatmap and PCA were obtained for the 3 different time points. At t1, the resulting clustering heatmap (**Figure 4A**) showed that group A4WS clustered with control groups. PCA also revealed a well-defined cluster with the control groups and a heterogeneous distribution of the water stress groups according to the genotype (**Figure 4B**). Samples from genotype A4 under stress (A4WS) were clustered close to the respective control group, whereas samples from other genotypes were clustered distant from the control groups. This difference was more evident in genotype A1 under water stress (A1WS). Principal component (PC) 1 contributed 90.1% of the total variance, with parameters A, gs and E as the top contributors for PC1. Overall, these results indicate response to drought is genotype-dependent. Genotype A4 is less sensitive to drought conditions and able to maintain normal net CO_2_ assimilation rates. In contrast, net CO_2_ assimilation rates in genotype A1 dropped on plants under drought. In contrast, samples from t2 are primarily clustered according to treatments, mainly due to physiological parameters, has shown in the clustering heatmap (**Figure 4C**). Moreover, samples clustered according to treatments as also shown by PCA scores plot that revealed two distinct groups: control samples (WW) and drought samples (WS) for the four genotypes (**Figure 4D**). Overall, these results indicate that although physiological performance on genotype A4 is affected after a more prolonged stress, plants are still able to maintain high levels of net CO_2_ assimilation rates, in contrast with the other genotypes tested. Finally, a clustering heatmap (**Figure 4E**) where genotype A1 was grouped on a separate cluster was obtained for t3. These results were confirmed by a PCA (**Figure 4F**) that showed three distinct groups: (1) control and recovered plants from genotypes A2, A3 and A4, (2) control plants from genotype A1, and (3) recovered plants from genotype A1, revealing a distinct behaviour of recovered plants from genotype A1.

### 3.2 Metabolomics

**Figure 5:**
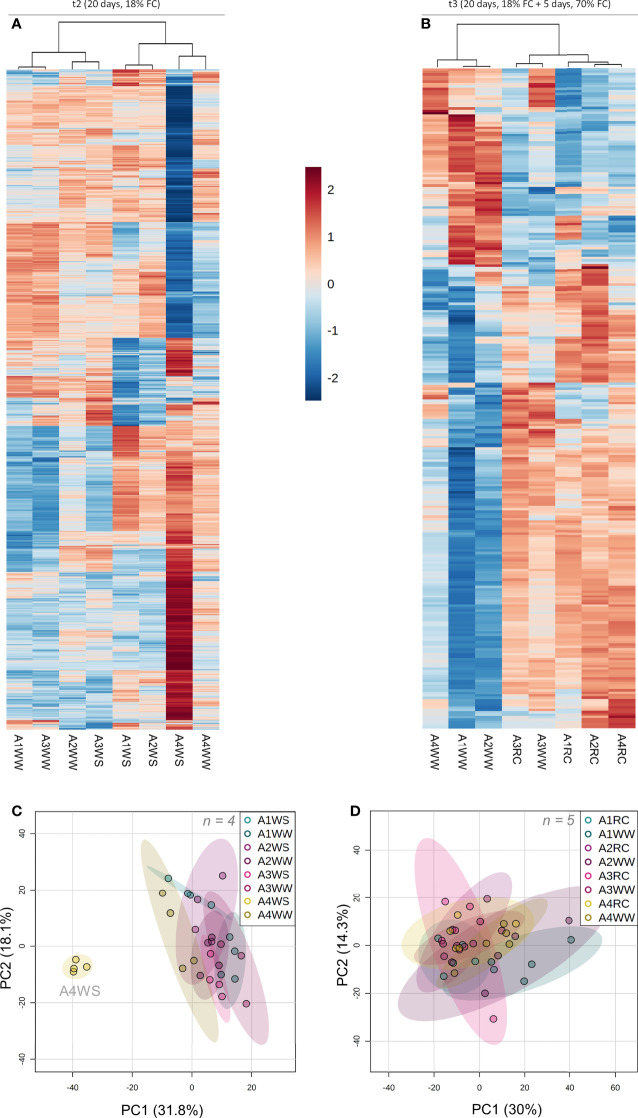
Untargeted metabolomic analysis on t2 (20 days) and t3 (20 + 5 days) on 4 genotypes (A1, A2, A3 and A4) and 3 water regimes (well-watered, water-stress and recover): heat map representing top 500 metabolites significantly different (p < 0.01) between groups (group is indicated at the bottom of the figure) on t2 **(A)** and t3 **(B)**, principal component analysis with all the metabolites on t2 **(C)** and t3 **(D)**. Data was normalised by median, cube root transformed and Pareto-scaled (n = 4-5).

To obtain a global overview of the metabolic shifts underpinning drought resistance in *A. unedo*, a LCMS untargeted metabolomics was carried out as previously described (Luna et al., 2020). MSDIAL processing of 12 282 detected raw features yielded a total of 3953 filtered metabolomics variables, of which 74 were identified as known metabolites based on publicly available databases (Tsugawa et al., 2015), whereas the remaining features were unknown metabolites. The identified metabolites include abscisic acid, lipids, terpenes, methoxyphenols, flavonoids and flavonoid glycosides (**Table S1**). Subsequent clustering of the 1590 most significant features (p < 0.01) revealed a specific metabolic profile of genotype A4 under water stress (**Figure 5A**). Based on the relative concentration of 3953 metabolic features, a PCA was used to further understand the dynamic patterns of metabolite concentration under drought, which revealed 2 distinctive clusters: (i) A4WS samples, and (ii) drought samples from the other three genotypes and all the control samples (**Figure 5B**). Although differences in metabolic markers were found between WW and RC groups, as revealed by clustering heatmap (**Figure 5C** and **Table S3**), samples were clustered relatively close together by PCA (**Figure 5D**).

**Figure 6:**
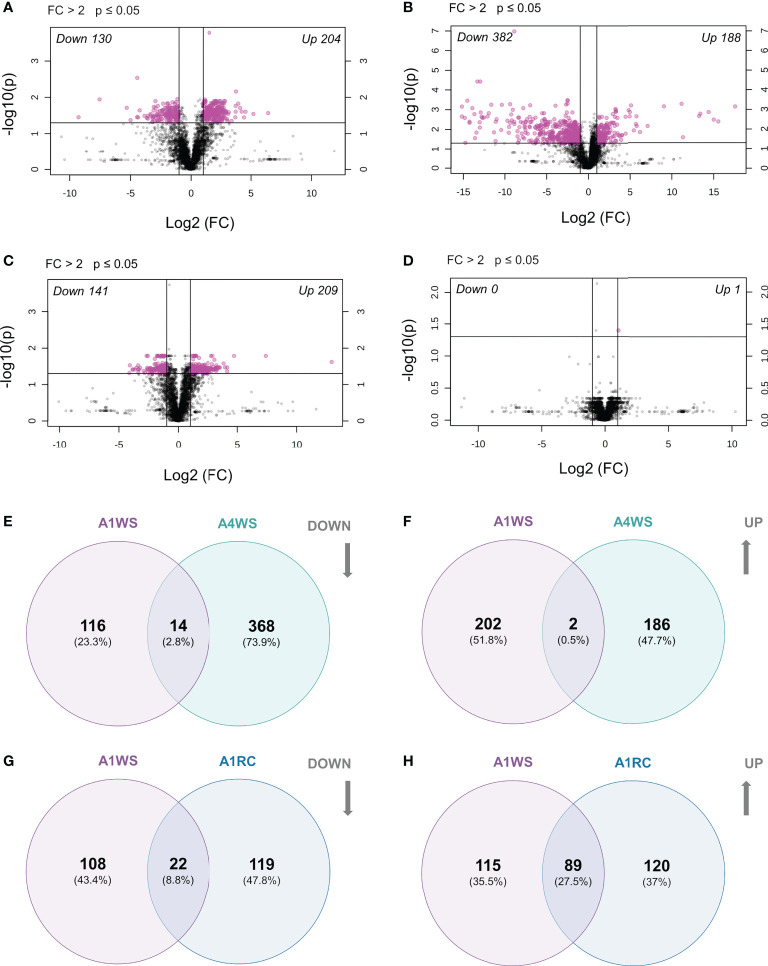
Differentially expressed metabolites on t2 (20 days) and t3 (20 + 5 days) on 2 genotypes (A1 and A4) and 3 water regimes (well-watered, water-stress and recover): volcano plot for genotype A1 indicating significantly (p < 0.05 and FC > 2) up- and down-regulated metabolites between treatments on t2 **(A)** and t3 **(B)**, volcano plot for genotype A4 indicating significantly (p < 0.05 and FC > 2) up- and down-regulated metabolites between treatments on t2 **(C)** and t3 **(D)**, Venn diagram with the down-regulated metabolites shared between genotype A1 and A4 on water-stress treatments **(E)**, Venn diagram with the up-regulated metabolites shared between genotype A1 and A4 on water-stress treatments **(F)**, Venn diagram with the down-regulated metabolites shared between recover and water-stress treatments on genotype A1 **(G)**, Venn diagram with the up-regulated metabolites shared between recover and water-stress treatments on genotype A1 **(H)**. Data was normalised by median, cube root transformed and Pareto-scaled (n = 4-5).

To search for the metabolites with statistical differences and according to fold change between treatments, a volcano plot (FC>2.0 and p-value<0.05) was constructed for each genotype, which revealed 334 differently accumulated metabolic markers between WW and WS treatments in genotype A1 (130 reduced and 204 increased) (**Figure 6A**). In genotype A4, 570 metabolic markers changed between WW and WS treatments (382 reduced and 188 increased) (**Figure 6B** and **Table S2**). Considering genotype A2 only two metabolites were found to increase upon stress (**Figure S2A**), whereas no metabolic markers were up- and/or down-regulated on genotype A3 (**Figure S2B**).

A volcano plot (FC > 2 and p-value ≤ 0.05) revealed 350 metabolic markers that were differentially accumulated between WW and RC treatments (141 reduced and 209 increased) (**Figure 6C**). On the other hand, the volcano plot showed only one up-regulated metabolic marker with statistical significance in genotype A4 (**Figure 6D**). A similar result was obtained for genotypes A2 and A3, with no metabolic markers found to have different concentrations (**Figures S2C, D**).

To identify the putative metabolites that are genotype-specific under drought, a Venn diagram was built to compare metabolic markers showing statistically significant differences between genotypes (A1 and A4). Only a few were found to be common between genotypes under drought: 14 metabolic markers were reduced in both genotypes (A1WS and A4WS), and only 2 were increased (**Figures 6E, F**). On the other hand, 116 and 368 metabolic markers were exclusively reduced in genotype A1 and A4, respectively, whereas 202 and 186 were increased solely (**Figures 6E, F**).

When the metabolic markers in genotype A1, that showed statistically significant differences between WW and RC treatments, were compared with WS treatment, only a few were found to be commonly down-regulated (22). Nevertheless, the common up-regulated metabolic markers were considerably higher (89) (**Figures 6G, H** and **Table S4**).

**Figure 7:**
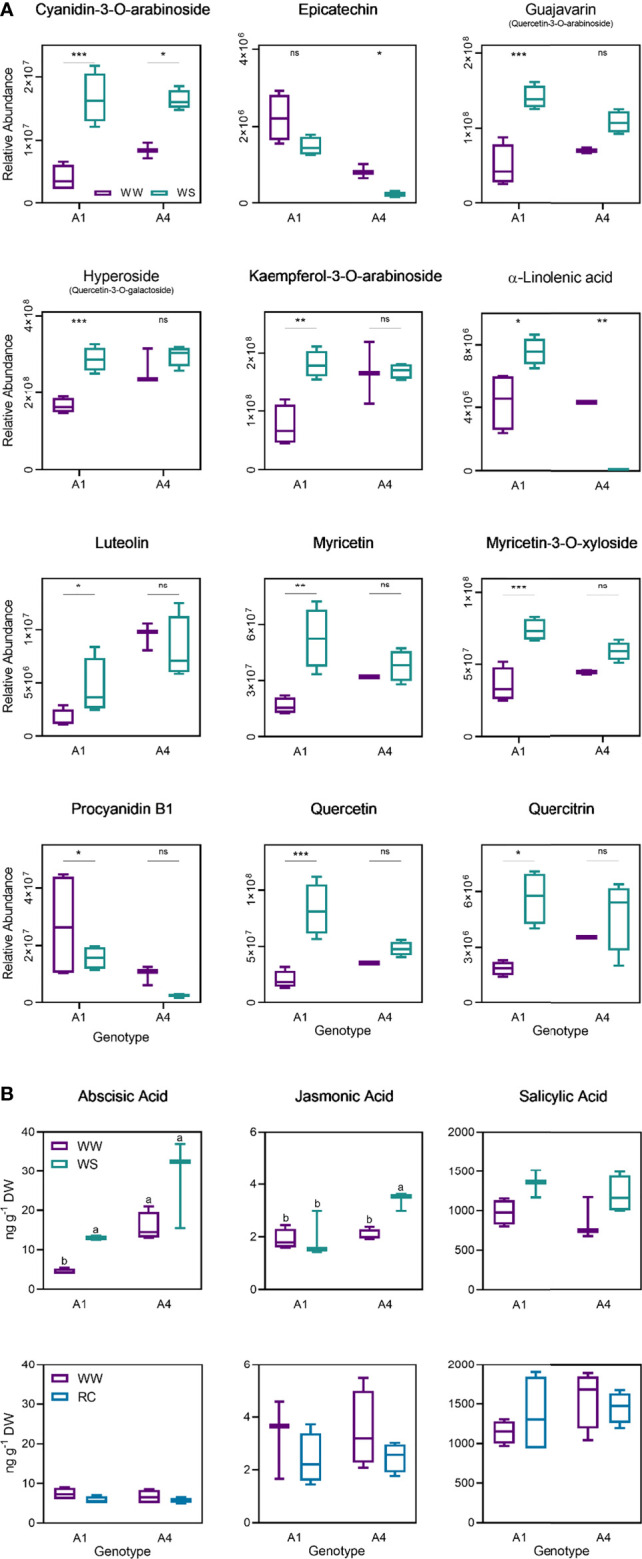
Relative abundance of cyanidin-3-O-arabinoside, epicatechin, guajavarin, hyperoside, kaempferol-3-O-arabinoside, α-linolenic acid, luteolin, myricetin, myricetin-3-O-xyloside, procyanidin B1m quercetin and quercitrin on well-watered and water stress groups from A1 and A4 genotypes on t2 (20 days) **(A)**, abscisic acid, jasmonic acid and salicylic acid on well-watered and water stress groups from A1 and A4 genotypes on t2 (20 days) and t3 (20 + 5 days) **(B)**. Means ± SDs, n = 3-4, different letters indicate significant differences between treatments at *P ≤* 0.05, according to a Tukey’s multi comparison test. * P value <0.05, ** P value <0.01, *** P value <0.001, ns, non significant (P > 0.05).

The differentially accumulated metabolic markers were putatively identified and included mainly phenols (**Tables S2, S4**). Some of the metabolites in genotype A1 that increased under stress are kaempferol, myricetin, quercetin and quercitrin (**Figure 7A**). In contrast, levels of α-linolenic acid, epicatechin and luteolin decreased in plants under water deficit in genotype A4 (**Figure 7A**). Levels of abscisic acid ranged from 4.5 ± 0.7 (ng g^-1^) to 13.0 ± 0.7 in genotype A1 between WW and WS groups, and from 15.8 ± 3.7 to 28.3 ± 11.3 in genotype A4 (**Figure 7B**). Although ABA concentration increased in both genotypes, statistically significant differences were found only for genotype A1. Levels of jasmonic acid increased from 2.1 ± 0.2 (ng g^-1^) to 3.4 ± 0.3 in genotype A4 between tested groups, and no differences were observed in genotype A1 (**Figure 7B**). Finally, although no statistical difference was obtained, the variation of salicylic acid levels in stressed plants was similar to those of ABA, with an increase in plants under water deficit (**Figure 7B**). Although a slight reduction of ABA and JA levels was observed on the recover plants (RC) in both genotypes, A1 and A4, no statistical differences were obtained (**Figure 7B**), and no differences were observed for salicylic acid concentration in the recovered group (**Figure 7B**). Overall, these results demonstrate that genotype A4 has a great phenotypic plasticity through metabolic regulation, whereas genotype A1 is a slower adapter.

**Figure 8:**
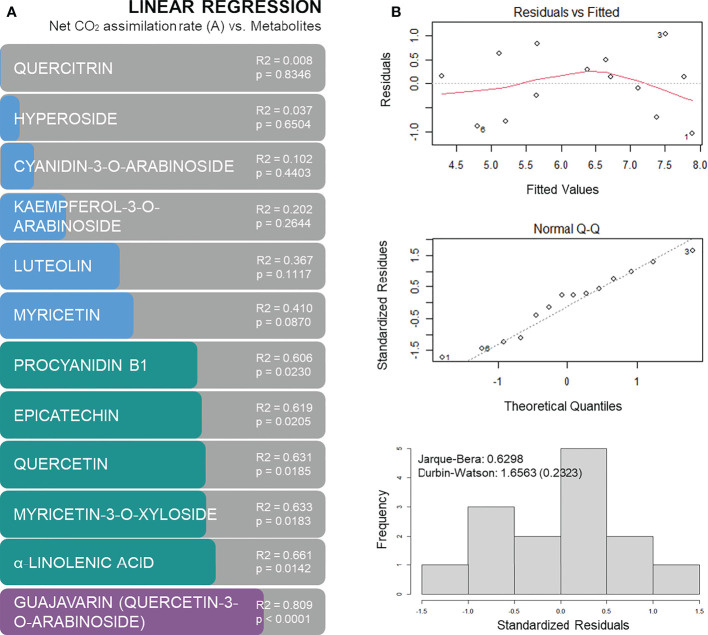
Regression models based on metabolites concentration to predict net CO_2_ assimilation rates: R^2^ of linear regression analysis with p-values **(A)**, and residuals versus fitted plot, Q-Q plot and histogram of residuals with Jarque-Bera and Durbin-Watson tests for linear regression between net CO_2_ assimilation and guajavarin (quercetin-3-O-arabinoside) concentrations **(B)**.

In order to predict the physiological performance of plants under drought based on metabolic markers, general linear models were calculated. From the identified metabolites that were found to be differently accumulated (p < 0.05) 12 were identified and tested for the construction of general linear models. Six of these metabolites provided a general linear model with a p-value below 0.05 and R^2^ above 0.6 (**Figure 8A**): procyanidin B1, epicatechin, quercetin, myricetin-3-O-xyloside, α-linolenic acid and guajavarin (quercetin-3-O-arabinoside). The best model was obtained for guajavarin with a R^2^ of 0.809 (p-value < 0.0001). The normality and independence of the residuals were confirmed in the model by a Q-Q plot, a distribution histogram, and the Jarque-Bera and Durbin-Watson tests (**Figure 8B**). Hence, metabolomics indicates that genotype A4 has a specific profile under drought, and subsequent machine learning predictions validate five phenols and a fatty acid as major and significant metabolic predictors.

## 4 Discussion

In this study, the effects of water stress on plant performance were analysed at different levels, including photosynthetic and plant water status, metabolomic shifts and hormonal dynamics. Our results pinpoint a differential plant response related to sampling time and water regime depending on the genotype tested.

The results presented in this analysis agree with our previous studies (2021b; Martins et al., 2019), and confirm a conservative response mechanism under water limitation scenarios, *i.e*. lower stomatal conductance, transpiration and net CO_2_ assimilation rates with higher intercellular CO_2_ concentrations. This conservative water use strategy, accomplished by stomatal closure, is typically exhibit by resprouter species such as *A. unedo*, to prevent embolism and hydraulic damage (Vilagrosa et al., 2014), which results in lower photosynthetic rates and CO_2_ accumulation. Our data clearly demonstrate that plant performance under water stress is genotype-dependent, which was also observed in other species, such as *Eucalyptus globulus* (Correia et al., 2014) and *Populus* × *euramericana* (Monclus et al., 2006). Genotype A4 not only showed better performance under water restriction but also when recovering, which indicates high phenotypic plasticity. Besides, the worst-performing genotype (A1) originates from a region with high average rainfall, whereas the best-performing genotype (A4) comes from an area with low average rainfall. This observation suggests a correlation between provenience and performance and the possible involvement of epigenetic modifications, such as DNA methylation, which are essential for trees to cope with rapidly changing environment (Kim et al., 2020; Sow et al., 2021). A previous study also correlated *A. unedo* plant provenience with performance under drought (Vasques et al., 2013), as seedlings from a driest region coped better with drought, which reinforces the idea of epigenetic involvement in drought tolerance. A similar observation was made by (Matías et al., 2016) when *Abies alba* from different geographic locations was under controlled stress conditions. Meijón et al. (2016) also established a connection between the metabolomic profile of *Pinus pinaster* genotypes and their region of origin. Nevertheless, in our study, this correlation was not found for all genotypes. Genotype A2, which comes from the region with lower average rainfall, harboured a poor performance similar to genotype A1. These data suggest that drought resistance is not always correlated with strawberry tree biogeographic history, which may be related to the high intraspecific diversity found by several authors in wild strawberry tree populations (Lopes et al., 2012; Gomes et al., 2013). A similar observation has been made by (Arend et al., 2011) on *Quercus* spp. populations. On a previous work carried out on strawberry tree from two different populations, plant performance under drought greatly varied between genotypes from the same provenience (Martins et al., 2021b), which confirms the results obtained in this study. Thus, although provenience is undoubtedly one of the aspects influencing plant performance under water deficit and should be considered for plant selection, genotype seems to be the key determining factor. Interestingly, Polle et al. (2019) suggested that a more flexible response to stress might be expected from trees rarely exposed to such conditions, which might be something worth to investigate in strawberry tree.

Associated with the physiological plasticity, metabolic adaptation is crucial in quickly changing environments. In strawberry tree, although no statistical differences were detected for most of the biochemical parameters, such as total phenols and flavonoids, a tendency of decrease was observed in the levels of chlorophyll and carotenoids in plants under drought and recovered from genotype A1, which might be due to a lower biosynthesis and/or higher degradation rates. The reduction of chlorophyll levels in droughted plants has already been reported due to water limitation (Fahad et al., 2017). In contrast, a tendency of increase of chlorophyll and carotenoid levels in recovered plants from genotype A4 indicates that its biosynthesis is augmented and that this genotype has a better recovery ability. An increase in anthocyanins levels was also observed in genotype A1, but only in the recovering plants. This increase might be related to the possible role of anthocyanins in the adaption of plants to stressful environments through a reduction in leaf light perception, causing a stress-resistant phenotype (Cirillo et al., 2021). A more detailed analysis proved the involvement of metabolites such as phenols in the drought resistance mechanism of *A. unedo*, as several metabolites were up- and down-regulated, especially in genotypes with a more contrast performance (*i.e.*, A1 and A4). In particular, genotype A4 presented a specific metabolic profile, which correlates with its better ability to perform under water stress, as shown by the physiological parameters. The metabolites that were putatively identified included phenols (flavonoids, flavonoid glycosides and metoxyphenols), lipids and terpenes. Flavonoids such as myricetin, quercetin and quercitrin were up-regulated in genotype A1 under water stress. Interestingly, the basal levels (*i.e.*, in the control group) of these phenols in genotype A4 were considerably higher when compared to basal levels in genotype A1, suggesting a predisposition of this genotype to cope with drought. These flavonoids have numerous roles, especially antioxidant, and seem to be key compounds in response under drought of Mediterranean plants (Laoué et al., 2022). Several studies related these flavonoids with drought response of plants: the accumulation of quercetin glycoside (2–3 fold increase) was observed in clover plants under drought (Nichols et al., 2015); in *Fraxinus ornus*, the accumulation of quercetin 3-O-glycosides under drought was associated with a reduction in ascorbate peroxidase and catalase activities (Fini et al., 2012), whereas a similar correlation between the accumulation of flavonoids like myricetin and antioxidant capacity was observed in *Populus deltoides* (Popović et al., 2016). These phenols (kaempferol, myricetin, quercetin and quercitrin) have already been identified in strawberry tree (Martins et al., 2021a), and a seasonal variation of their concentrations was observed, probably as a response to specific environmental conditions, which supports the contribution of such compounds in plant tolerance mechanisms to stress. Furthermore, this previous study identified chemotypes, thus confirming key genetic influences on metabolic responses. In terms of hormone shifts, here we report a considerable increase in the ABA levels in stressed plants of both genotypes. While a more pronounced increase occurred in genotype A1, which performed worst under water deficit, basal ABA levels in genotype A4 were considerably higher compared to genotype A1, which seems to confirm the pattern observed for phenols. Because this hormone also participates in other essential pathways, higher levels of ABA is a hallmark for plants under water deficit conditions (Sah et al., 2016). Other hormones like JA also increased in genotype A4, whereas a slight increase in SA concentrations was observed in our experiment, yet not statistically significant, possibly due to prolonged stress conditions that usually underpin the return to basal levels (Ali and Baek, 2020). Finally, satisfactory general linear models were obtained for procyanidin, epicatechin, quercetin, myricetin, α-linolenic acid and guajavarin (quercetin-3-O-arabinoside), which could be used to predict net CO_2_ assimilation rates. Previous works have successfully deployed machine learning approaches to predict complex phenotype traits such as relative growth rate or pathogen infections (Luna et al., 2020; Roch et al., 2020), so greater focus on this matter could produce interesting findings that facilitate the selection of drought tolerant tress on natural populations.

This study shows that sensitivity to water stress was highly genotype-dependent. The research also reports that physiological control seems to be the primary response mechanism of *A. unedo* under water stress, with a trade-off between transpiration water loss and CO_2_ assimilation. Furthermore, the recovery ability is also genotype-dependent, and in general, genotypes with better performance under stress also have an excellent recovery ability. This investigation also shows that plant provenience is an essential factor, but genotype is factually the key factor governing plant ability to cope with drought. Finally, one of the more significant findings from this study is the relevance of metabolomic changes to tackle drought stress, clearly supported by the current findings. Our data seems to indicate that plants following this efficient strategy are predisposed to drought, as several metabolites were found in high concentrations in control groups. Besides the genotype influence, this might be putatively related to the plant provenance and a pre-conditioning. The insights gained from this study are of particular interest to plant selection and breeding as they confirm previous data that pointed to a selection based on a single population, thus focused on the intra- rather than inter-specific diversity. Although these findings shed new light on strawberry tree drought tolerance mechanisms, some questions remain unaddressed. Plant geographic provenance and the possible benign effect of previous exposure to stress is still not clear. Thus, the exploration of epigenetics for drought improvement would be a fruitful area for further work (Kim et al., 2020; Sow et al., 2021). Another natural progression of this work would be to perform a targeted analysis on a broad range of metabolites to comprehend better the metabolic pathways involved in response to drought. Some metabolites might emerge as reliable predictors of plant performance under drought and be used as selection markers (Fernandez et al., 2021).

## Data availability statement

The original contributions presented in the study are included in the article/**Supplementary Material**. Further inquiries can be directed to the corresponding author.

## Author contributions

JM, GP, JC: experimental design. JM, AF and PM: experimental performance and data collection. JM and PP: data analysis. JM: manuscript writing. PP, AG-C, GP and JC: manuscript revision and edition. PP, AG-C, GP and JC: funding acquisition. All authors contributed to the article and approved the submitted version.

## Funding

Foundation for Science and Technology (Portugal) supported JM PhD fellowship (SFRH/BD/122478/2016). This work was carried out at the R&D Unit Center for Functional Ecology - Science for People and the Planet (UIDB/04004/2020), and CESAM (UID/50017/2020 + UIDB/50017/2020) financed by FCT/MCTES through national funds (PIDDAC). FCT also supported PM (SFRH/BD/143879/2019). The authors also acknowledge ReNATURE project (CENTRO-01-0145-FEDER-000007), F4F-Forest for the future (CENTRO-08-5864-FSE-000031) and CULTIVAR project (CENTRO-01-0145-FEDER-000020), co-financed by the Regional Operational Programme Centro 2020, Portugal 2020, and the European Union, through the European Fund for Regional Development (ERDF). The authors are grateful for financial support from MetaboHUB (ANR-11-INBS-0010) and PHENOME (ANR-11-INBS-0012) projects for metabolomic analyses.

## Conflict of interest

The authors declare that the research was conducted in the absence of any commercial or financial relationships that could be construed as a potential conflict of interest.

## Publisher’s note

All claims expressed in this article are solely those of the authors and do not necessarily represent those of their affiliated organizations, or those of the publisher, the editors and the reviewers. Any product that may be evaluated in this article, or claim that may be made by its manufacturer, is not guaranteed or endorsed by the publisher.

## Supplementary material

Supplementary Figure 1 Average rainfall (mm) from 1970 to 2019 in seven meteorological stations across continental Portugal and location of the four mother plants used for micropropagation **(A)** and water irrigation conditions, analysis carried out and sample size used on the experiment along the three time points sampled: t1 (10 days stress), t2 (20 days stress) and t3 (20 days stress plus 5 days recover) **(B)**.

Supplementary Figure 2 Metabolomic analysis on genotypes A2 and A3 at t2 **(A-B)** and t3 **(C-D)**: volcano plot for genotype A2 indicating significantly (p < 0.05 and FC > 2) up- and down-regulated metabolites on water stress group when compared to well-water group **(A)**, volcano plot for genotype A3 indicating significantly (p < 0.05 and FC > 2) up- and down-regulated metabolites on water stress group when compared to well-water group **(B)**, volcano plot for genotype A2 indicating significantly (p < 0.05 and FC > 2) up- and down-regulated metabolites on water stress group when compared to recover group **(C)**, volcano plot for genotype A3 indicating significantly (p < 0.05 and FC > 2) up- and down regulated metabolites on water stress group when compared to recover group **(D)**. Data was normalised by median, cube root transformed and Pareto-scaled (n = 4-5).
