# Supplementary figures and images for: Genotype determines Arbutus unedo L. physiological and metabolomic responses to drought and recovery
Source: Front Plant Sci. 2022 Nov 22;13:1011542. doi: 10.3389/fpls.2022.1011542 (PMC9723149; doi:10.3389/fpls.2022.1011542)

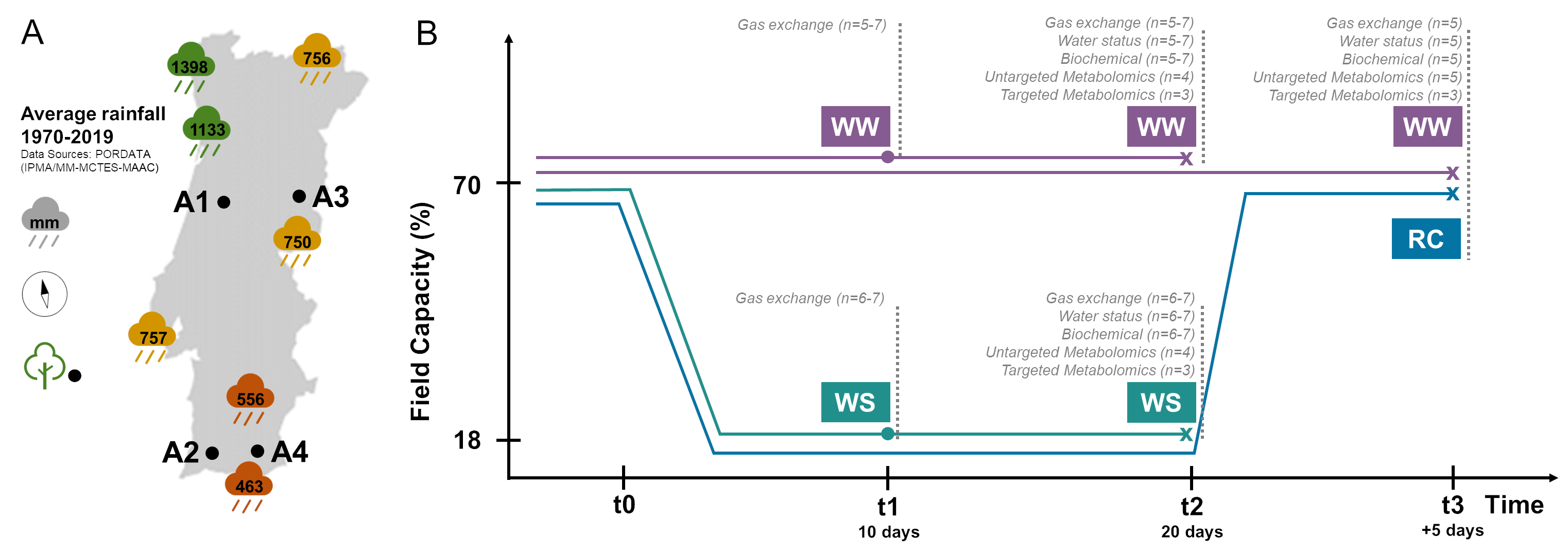

Supplement: Supplementary Figure 1 — Average rainfall (mm) from 1970 to 2019 in seven meteorological stations across continental Portugal and location of the four mother plants used for micropropagation (A) and water irrigation conditions, analysis carried out and sample size used on the experiment along the three time points sampled: t1 (10 days stress), t2 (20 days stress) and t3 (20 days stress plus 5 days recover) (B). [file Image_1.tif]

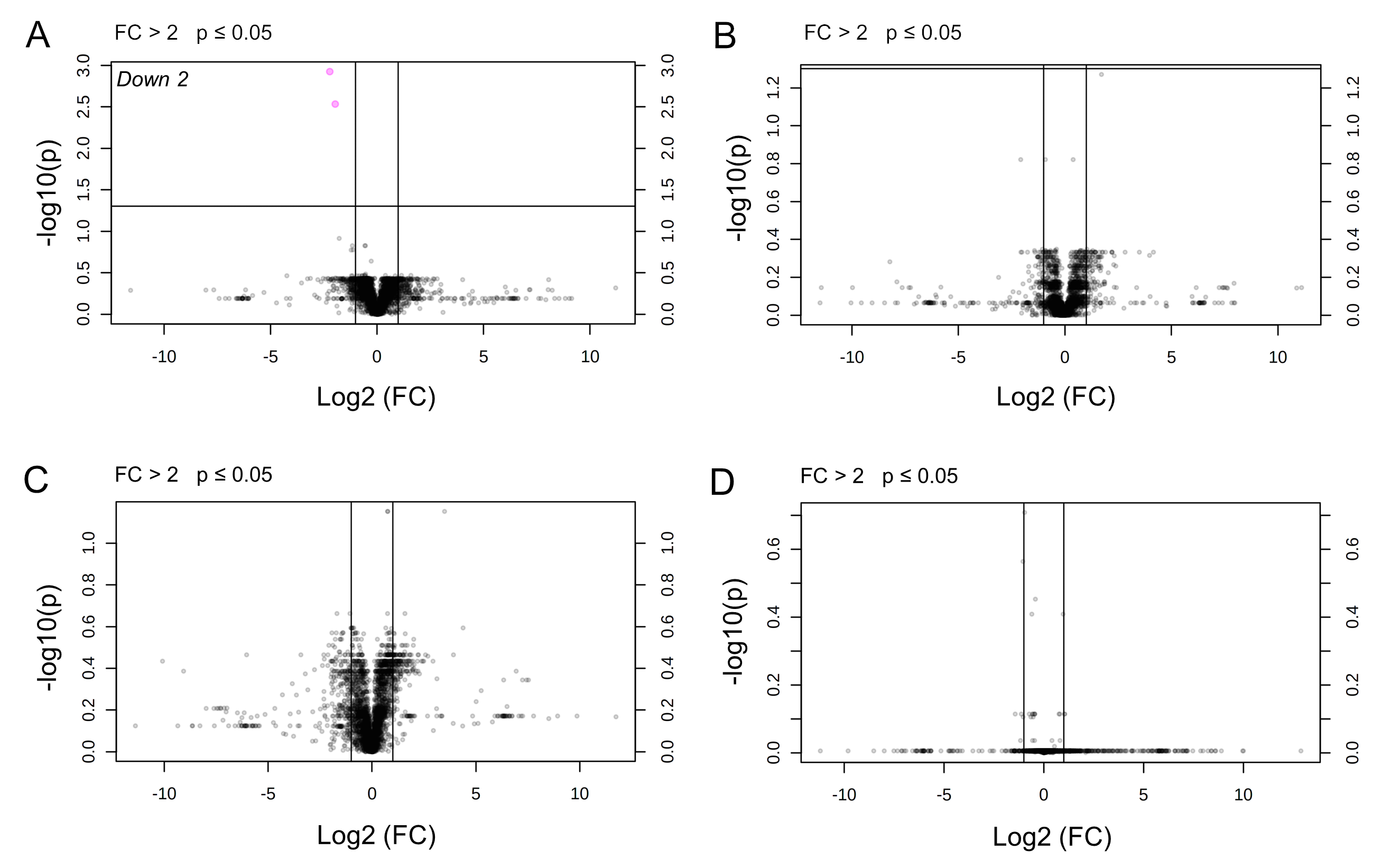

Supplement: Supplementary Figure 2 — Metabolomic analysis on genotypes A2 and A3 at t2 (A-B) and t3 (C-D): volcano plot for genotype A2 indicating significantly (p < 0.05 and FC > 2) up- and down-regulated metabolites on water stress group when compared to well-water group (A), volcano plot for genotype A3 indicating significantly (p < 0.05 and FC > 2) up- and down-regulated metabolites on water stress group when compared to well-water group (B), volcano plot for genotype A2 indicating significantly (p < 0.05 and FC > 2) up- and down-regulated metabolites on water stress group when compared to recover group (C), volcano plot for genotype A3 indicating significantly (p < 0.05 and FC > 2) up- and down regulated metabolites on water stress group when compared to recover group (D). Data was normalised by median, cube root transformed and Pareto-scaled (n = 4-5). [file Image_2.tif]
